# Therapeutic Antiviral Effect of the Nucleic Acid Polymer REP 2055 against Persistent Duck Hepatitis B Virus Infection

**DOI:** 10.1371/journal.pone.0140909

**Published:** 2015-11-11

**Authors:** Faseeha Noordeen, Catherine A. Scougall, Arend Grosse, Qiao Qiao, Behzad B. Ajilian, Georget Reaiche-Miller, John Finnie, Melanie Werner, Ruth Broering, Joerg F. Schlaak, Andrew Vaillant, Allison R. Jilbert

**Affiliations:** 1 Department of Molecular and Cellular Biology, School of Biological Sciences, University of Adelaide, Adelaide, SA, Australia; 2 Department of Microbiology, Faculty of Medicine, University of Peradeniya, Peradeniya, Sri Lanka; 3 SA Pathology, Hanson Institute, Centre For Neurological Diseases, Adelaide, SA, Australia; 4 Department of Gastroenterology and Hepatology, University Hospital, University of Duisburg-Essen, Essen, Germany; 5 Replicor Inc., Montreal, Quebec, Canada; Indiana University, UNITED STATES

## Abstract

Previous studies have demonstrated that nucleic acid polymers (NAPs) have both entry and post-entry inhibitory activity against duck hepatitis B virus (DHBV) infection. The inhibitory activity exhibited by NAPs prevented DHBV infection of primary duck hepatocytes *in vitro* and protected ducks from DHBV infection *in vivo* and did not result from direct activation of the immune response. In the current study treatment of primary human hepatocytes with NAP REP 2055 did not induce expression of the *TNF*, *IL6*, *IL10*, *IFNA4* or *IFNB1* genes, confirming the lack of direct immunostimulation by REP 2055. Ducks with persistent DHBV infection were treated with NAP 2055 to determine if the post-entry inhibitory activity exhibited by NAPs could provide a therapeutic effect against established DHBV infection *in vivo*. In all REP 2055-treated ducks, 28 days of treatment lead to initial rapid reductions in serum DHBsAg and DHBV DNA and increases in anti-DHBs antibodies. After treatment, 6/11 ducks experienced a sustained virologic response: DHBsAg and DHBV DNA remained at low or undetectable levels in the serum and no DHBsAg or DHBV core antigen positive hepatocytes and only trace amounts of DHBV total and covalently closed circular DNA (cccDNA) were detected in the liver at 9 or 16 weeks of follow-up. In the remaining 5/11 REP 2055-treated ducks, all markers of DHBV infection rapidly rebounded after treatment withdrawal: At 9 and 16 weeks of follow-up, levels of DHBsAg and DHBcAg and DHBV total and cccDNA in the liver had rebounded and matched levels observed in the control ducks treated with normal saline which remained persistently infected with DHBV. These data demonstrate that treatment with the NAP REP 2055 can lead to sustained control of persistent DHBV infection. These effects may be related to the unique ability of REP 2055 to block release of DHBsAg from infected hepatocytes.

## Introduction

Hepatitis B virus (HBV) has affected more than 2 billion people worldwide, leaving more than 360 million of these individuals with chronic HBV (CHB) infection [1]. Treatment with immunotherapies like pegylated interferon can achieve control of CHB in only a small fraction of patients and HBV polymerase inhibitors like entecavir (ETV) and tenofovir disoproxil fumarate inhibit HBV DNA replication and decrease levels of HBV DNA in the blood but rarely lead to the clearance of CHB infection [2–6]. As such, there is an urgent need for more effective treatments for CHB infection.

Nucleic acid polymers (NAPs) are phosphorothioated oligonucleotides (PS-ONs) whose biological activity is derived from their sequence independent properties as amphipathic polymers, a functionality which is distinct from sequence dependent properties of antisense oligonucleotides. NAPs bind to uncomplexed amphipathic α-helices in class I surface glycoproteins [7], structures which are conserved in the surface glycoproteins of many enveloped viruses [8, 9]. This is the target interaction that underlies the broad spectrum activity of NAPs against many enveloped viruses including human immunodeficiency virus, cytomegalovirus, herpes simplex virus 1 and 2 and lymphochoriomeningitis virus [7, 10–13]. In all of the above studies, the antiviral effect did not result from any direct immunostimulation and was strictly dependent on the presence of phosphorothioation, which imparts an increased amphipathic character to NAPs [14]. The antiviral effect was also dependent on polymer size: only NAPs greater than 30 nucleotides in length displayed significant antiviral activity while NAPs 20 nucleotides and lower had no antiviral effect. These unique determinants of NAPs antiviral activity are consistent with the large target interface between NAPs and amphipathic protein domains.

NAPs have also been shown to inhibit a post-binding cell entry step that prevents infection with the class 2 virus, hepatitis C virus (HCV) [15] and this antiviral activity was shown to have the same phosphorothioation and size dependent activity as observed with class 1 viruses. However, the E glycoprotein in HCV contains no amphipathic structure similar to those common in class 1 viral fusion glycoproteins and it has been suggested that the antiviral activity of NAPs against HCV infection is not mediated through interaction with the viral glycoprotein but through an amphipathic interaction with a cellular component involved in HCV entry [15].

NAPs have also been shown to have entry and post-entry inhibitory activity against another class 2 enveloped virus, the duck hepatitis B virus (DHBV). NAPs were shown not to be hepatotoxic in primary duck hepatocytes (PDH) and the structure-activity relationship of the antiviral activities of NAPs against DHBV infection of PDH was strictly dependent on the presence of phosphorothioation and polymer size [16] and demonstrated that NAPs act independently of immunostimulation *in vitro and in vivo* [16, 17]. Importantly NAPs were shown to have a unique post-entry inhibitory activity against DHBV infection which appears to be essential for activity *in vivo*, while the entry blocking activity appears to be dispensable [17]. Although the mechanism of action of post-entry inhibitory activity of NAPs in DHBV infection has not yet been elucidated, these studies demonstrated that NAPs had a potent antiviral effect *in vivo*.

In the current study, the NAP REP 2055 was first tested for immunostimulatory activity in primary human hepatocytes (PHH) *in vitro* and was then assessed for its ability to treat pre-established, persistent DHBV infection *in vivo*, firstly in a preliminary experiment (Experiment 1) using different dosing regimens of REP 2055, followed by a larger second experiment (Experiment 2), where ducks with persistent DHBV infection were treated for 28 days with REP 2055 or normal saline (NS). The studies were performed to examine the potential of NAP 2055 as a new therapeutic approach for CHB infection in humans.

## Materials and Methods

### Synthesis of REP 2055

REP 2055 (REP 9AC) is a phosphorothioated oligodeoxynucleotide (PS-ON) with the sequence (dAdC)_20_ and was prepared as previously described [15]. Lyophylized REP 2055 was re-dissolved in NS at a concentration of 10 mg/mL and filter sterilized prior to intraperitoneal (IP) injection into ducks. For *in vitro* stimulation, lyophylized REP 2055 was re-dissolved in phosphate buffered saline at a concentration of 13.5 mg/mL and filter sterilized.

### Stimulation of PHH with REP 2055

PHH were prepared using liver samples obtained after tumour resection (n = 3). The liver tissues were perfused and digested using two-step collagenase perfusion as described elsewhere [18]. Informed consent in writing was obtained from each patient, and the work was approved by the Institutional Review Board (Ethics Committee) of the Faculty of Medicine at the University Duisburg-Essen. Hepatocytes were seeded into collagen-I-coated culture plates using DMEM Ham’s F12 (PAA, Pasching, Austria) supplemented with 10% FCS (PAA), 1% L-glutamine (PAA) and 0.08 U/mL penicillin/streptomycin (PAA). PHH were cultured for 24 h, the medium was changed and cells were treated with different concentrations of REP 2055 for 6 hr. Immune stimulatory controls were used to indicate the responsiveness of PHH: ODN2216 (2 μM, Invivogen, Toulouse, France), Pam3CK4 (1μM, Invivogen) polyinosinic:polycytidylic acid (polyI:C, 25 μg/ml, Invivogen). Total RNA was isolated using the Qiazol™ and the RNeasy Mini Kit (Qiagen, Hilden, Germany). Quantitative RT-PCR was performed with the QuantiTect SYBR Green RT-PCR Kit (Qiagen) using 0.1–0.3 μg of total RNA. Gene expression of *IFNA4*, *IFNB1*, *TNF*, *IL6*, and *IL10* was determined using commercially available primer sets (QuantiTec Primer Assay, Qiagen). Calculated copy numbers were normalized to beta actin (NM 001101.30), detected with forward primer 5’-TCC CTG GAG AAG AGC TAC GA-3’ and reverse primer 5’AGC AAT GTG TTG GCG TAC AG-3’ [18)]

### Animal ethics statement

All animal handling protocols and operating procedures were approved by the Animal Ethics Committee of SA Pathology and the University of Adelaide and adhered to the standards of the National Health and Medical Research Council of Australia.

### DHBV infection

Pekin Aylesbury ducks (*Anas platyrhynchos)* were obtained at day 1 post-hatch from a commercial poultry supplier. All ducks were held at the SA Pathology animal house. Fourteen-day-old ducks were infected with DHBV as previously described [19] by intravenous (IV) inoculation with 5x10^8^ DHBV genome equivalents via the jugular vein. This protocol results in rapid spread of DHBV infection in the liver and invariably causes persistent DHBV infection [19–22].

### REP 2055 dosing *in vivo* Experiment 1

**Fig 1:**
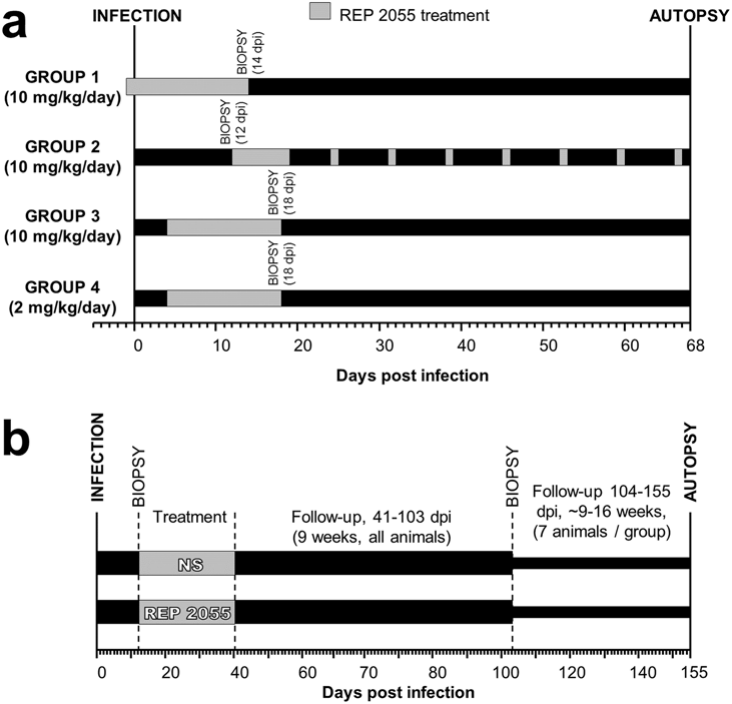
Experimental design of REP 2055 and NS treatment in *in vivo* Experiment 1 and 2. Fourteen-day-old ducks were infected with 5x10^8^ DHBV genome equivalents via the jugular vein. In Experiment 1 (a) all ducks were treated by IP injection with REP 2055. Group 1 received 10 mg/kg/day from 1 day prior to DHBV infection to 14 dpi; Group 2 received 10 mg/kg/day from 12–19 dpi and 10 mg/kg once weekly for 49 days; Group 3 received 10 mg/kg/day from 4–18 dpi and; Group 4 received 2 mg/kg/day from 4–18 dpi. After treatment, ducks in Groups 1, 3 and 4 were followed for an additional 49 days, from 19–68 dpi. Liver biopsies were performed prior to treatment in Group 2 and at the end of treatment in Groups 1, 3 and 4. Autopsies were performed at 68 dpi at the end of treatment in Group 2 and the end of follow-up in Groups 1, 3 and 4. In Experiment 2 (b), 14 DHBV-infected ducks were treated by IP injection with 10 mg/kg/day of REP 2055 from 12–40 dpi. A control group of 14 DHBV-infected ducks received daily IP injections of NS. Blood samples were collected during treatment and from 41–103 dpi during the first 9 weeks of follow-up. Based on interim analysis of serum DHBV DNA, 7 REP 2055-treated ducks that maintained control of their infection and 7 randomly selected NS-treated ducks, were followed from 103–155 dpi (total 16 weeks of follow-up). Liver biopsies were performed in all animals at 12 dpi prior to treatment. Biopsies or autopsies were performed at 103 dpi (9 weeks follow-up) and additional autopsies were performed in 7 animals per group at 155 dpi (16 weeks follow-up).

Four groups of 5 ducks were treated with REP 2055 via IP injection as follows (Fig 1A): Group 1 received 10 mg/kg/day from 1 day prior to DHBV infection to 14 days post-infection (dpi); Group 2 received 10 mg/kg/day from 12–19 dpi and 10 mg/kg once weekly thereafter for 49 days (similar to the dosing regimen used for PS-ONs in humans); Group 3 received 10 mg/kg/day from 4–18 dpi and; Group 4 received 2 mg/kg/day from 4–18 dpi. Ducks in Groups 1, 3 and 4 were followed for an additional 49 days, from 19–68 dpi. Duck 289 (Group 3) died at 10 dpi and duck 294 (Group 4) did not recover from anaesthesia during the biopsy at 16 dpi. Neither event was related to REP 2055 treatment. Blood samples were collected during treatment and follow-up and were used for detection of DHBsAg by ELISA and DHBV DNA extraction for qPCR as described below. Liver biopsies were performed prior to treatment in Group 2 and at the end of treatment in Groups 1, 3 and 4. Autopsies were performed at 68 dpi, corresponding to the end of treatment in Group 2 and the end of follow-up in Groups 1, 3 and 4 (Fig 1A).

### REP 2055 dosing *in vivo* Experiment 2

Fourteen DHBV-infected ducks were treated by IP injection with 10 mg/kg/day of REP 2055 from 12–40 dpi (Fig 1B). A control group of 14 DHBV-infected ducks received daily IP injections of equivalent volumes of NS. Blood samples were collected during treatment and from 41–103 dpi during the first 9 weeks of follow-up. Based on interim analysis of serum DHBV DNA, 7 REP 2055-treated ducks that maintained control of their infection, and 7 randomly selected NS-treated ducks, were followed from 103–155 dpi (total of ~ 16 weeks of follow-up) (Fig 1B). Blood samples were used for complete blood examination (CBE), quantitation of the liver enzymes, γ glutamyl transferase (GGT), alanine amino transferase (ALT) and aspartate transferase (AST), detection of DHBsAg and anti-DHBV antibodies by ELISA and extraction and quantitation of DHBV DNA by qPCR. CBE and liver enzyme tests were performed by the IDEXX International Veterinary Laboratory Service, South Australia. Liver biopsies were performed in all animals prior to treatment at 12 dpi. Biopsies or autopsies were performed at 103 dpi (9 weeks follow-up) and additional autopsies were performed in 7 animals per group at 16 weeks of follow-up.

### Liver biopsy and autopsy of ducks

Liver biopsies were performed under anaesthesia as previously described [23] and consisted of 200–300 mg of liver tissue taken from a resection of the lower section of the right lobe of the liver. Ducks received 5 mg/kg of ketoprofen (Troy Laboratories Pty Ltd, Australia) via intramuscular injection as a post-operative analgesia. For autopsies, ducks were overdosed with an anaesthetic (Lethobarb®, Virbac; 100 mg/kg) via the jugular vein. Biopsy and autopsy liver tissues were snap-frozen prior to storage at -80°C or fixed in 10% neutral buffered formalin in PBS overnight or in ethanol:acetic acid (EAA, 3:1 v/v) for 30 min and then transferred to 70% cold ethanol. Formalin- and EAA-fixed tissues were then wax-embedded and sectioned at 6 μm at the Hanson Institute Centre for Neurological Diseases, SA Pathology.

### Immunostaining of DHBV surface antigen (DHBsAg) and core antigen (DHBcAg)

DHBsAg positive hepatocytes were detected as previously described [23, 24]. In brief, EAA-fixed liver sections were de-waxed in xylene (Merck) and rehydrated through ethanol to PBS and then treated with 0.5% H_2_O_2_ (BDH Chemicals) in PBS to inactivate endogenous tissue peroxidases. After washing in PBS, sections were blocked with normal sheep serum (NSS) then incubated with primary anti-DHBV preS monoclonal antibodies, 1H1 [25] and secondary horseradish peroxidase (HRP)-conjugated sheep anti-mouse IgG (GE Healthcare Ltd, Cat #NA931V). Bound HRP was visualized by treating slides with diaminobenzidine tetrahydrochloride (Sigma Aldrich) and then sections were counter-stained with haematoxylin, dehydrated in ethanol followed by xylene and mounted in Entellan medium (Merck).

A similar procedure was followed for the immunostaining of formalin-fixed sections to identify DHBcAg positive hepatocytes, with the following modifications. After de-waxing and rehydration, antigen retrieval was performed by heating in a microwave for 10 min in 10 mM sodium citrate buffer. The blocking reagent contained 10% normal duck serum (NDS), 10% foetal bovine serum (FBS) and 10% normal goat serum (NGS) in PBS. Primary anti-DHBcAg polyclonal antibodies (Devin Teoh, personal communication), were used at a dilution of 1:400 in the blocking reagent (as described above), followed by secondary, HRP-labelled goat anti-rabbit IgG (KPL, Cat # 074–1506) at a dilution of 1:200. Bound HRP was then visualised as described above.

Counts of DHBsAg and DHBcAg positive hepatocytes were performed at 40x magnification, with the aid of an eyepiece graticule (250 x 250 μm). For DHBsAg detection a minimum of 355 fields (containing ~100,000 hepatocytes) were scanned in each liver section and expressed as a percentage of total haematoxylin stained hepatocyte nuclei yielding a minimum sensitivity of detection of 0.001%. DHBcAg positive hepatocytes were counted in a similar manner, but the smaller size of the tissue pieces fixed in formalin resulted in a minimum sensitivity of detection of 0.006%. Tissue sections were photographed using 20x magnification and AnalySIS FIVE (Olympus Soft Imaging System).

### Detection of DHBsAg by ELISA

Serum samples were tested to determine levels of DHBsAg by ELISA as previously described [23]. DHBsAg levels were determined using a standard curve generated using serial dilutions of a pool of serum collected from congenitally DHBV-infected ducks (Pool 8) containing 50 μg/ml of DHBsAg [26]. The positive cut-off reading was determined as the value obtained using NDS tested in duplicate plus 2 standard deviations (SD). Serum samples, diluted 5-fold starting at a dilution of 1/100, were used to coat 96-well microtitre plates. DHBsAg was detected using primary anti-DHBV preS monoclonal antibodies, 1H1 [25] followed by secondary HRP conjugated sheep anti-mouse polyclonal antibodies (GE Healthcare UK Limited, Cat. # NA931V). Following three PBS washes, 100 μl of o-phenylenediamine dihydrochloride (OPD) HRP substrate solution (Sigma-Fast™, Sigma-Aldrich, Germany) was added to each well and incubated for 15 min in the dark followed by the addition of 50 μl of 2.5 M H_2_SO_4_ to stop the reaction. Optical density (OD) values were read using Spectra Max M2 plate reader (Millennium Science, USA) at 490 nm and results were analysed using GraphPad Prism software.

### Detection of anti-DHBs and anti-DHBc antibodies by ELISA

Costar 3590 flat-bottomed 96-well microtitre plates were coated with anti-DHBV preS monoclonal antibodies [25] in 0.1 M NaHCO_3_ (pH = 9.6) at 37°C for 1 hr followed by 4°C overnight. The plates were washed in PBS containing 0.05% tween-20 (PBS-T) and then coated with skim milk PBS-T to block any non-specific binding sites. Plates were again washed in PBS-T and then coated with 100 μl of sucrose-purified DHBsAg (1 ng/μl) [23] in PBS-T and incubated at 37°C for 1 hr. For detecting anti-DHBc antibodies, Costar 3590 plates were coated with 100 μl of purified recombinant DHBcAg [27] at a concentration of 1 μg/ml in PBS. After the PBS-T wash, the plates for both assays were coated with duplicate 5-fold dilutions of individual test serum samples. A positive control consisted of duck serum containing high titre anti-DHBs and anti-DHBc antibodies. NDS was used as a negative control and the average of quadruplicate NDS OD values were used set an OD cut off value. Serum samples were incubated at 37°C for 1 hr then all plates were washed in PBS-T. Bound antibodies were then detected in both assays by sequential incubation with rabbit anti-duck IgY polyclonal antibodies [23] and HRP-conjugated goat anti-rabbit polyclonal antibodies (KPL, Cat.# 074–1506). Washes between all steps were performed using an automated plate washer (ELx405 Select, BioTek^®^). Bound HRP was detected using OPD substrate as described above. OD values were analysed using MS Excel and regression analysis was used to interpolate the dilution of serum required to achieve the OD value obtained with NDS.

### Detection of DHBV DNA in liver tissue by qPCR and Southern blot hybridisation

Extraction of total cellular DNA was performed using a DNeasy kit (Qiagen) as per the manufacturer’s instructions. Liver DNA samples were digested with EcoRI and 150 ng aliquots were used in qPCR assays to detect DHBV total and cccDNA as previously described [28]. Standard curves were established using EcoRI-digested pBL4.8x2 [24, 28, 29] diluted to contain 10^8^−10^1^ copies of DHBV DNA. The qPCR methods were validated to reliably detect DHBV DNA in the presence of REP 2055 in liver tissue and serum matricies using spiking control experiments (data not shown). Primers for total DHBV DNA were situated within the polymerase open reading frame; P1-5’CAGATCTCCCTCGCCTAGGA (nt 390–410), P2- 3’ATTGCCTCATGCTGCATCAC (nt 666–646) while primers for cccDNA spanned the cohesive overlap region; P3-5’CCTGATTGGACGGCTCTTAC (nt 2462–2481), P4- 3’AAAGGTACAGTCAAGGCTGA (nt 2618–2599). The qPCR was performed using SYBR green qPCR master mix (Applied Biosystems) and an AB StepOnePlus Real Time PCR machine. The qPCR conditions and the standard curve were set up with an initial denaturation step at 50°C for 2 min, activation of polymerase at 95°C for 10 min followed by 40 cycles of 15 sec at 95°C and 1 min at 60°C. Southern blot hybridisation was performed as previously described [27] using 2 μg liver DNA samples per well extracted using a DNeasy kit (Qiagen) and quantitated using an ND1000 spectrophotometer (Nanodrop, USA).

### Detection of DHBV DNA in serum by qPCR

DNA was isolated from 200 μl of serum using a Charge Switch viral nucleic acid extraction kit (Invitrogen) and was eluted in a volume 50 μl. Serum DHBV DNA was detected by qPCR using primers P1 and P2 described above again with SYBR Green PCR Master Mix and an AB StepOnePlus^TM^ Real Time PCR machine. Standard curves were established using dilutions of plasmid pBLDHBV4.8x2 [24, 28, 29] containing 10^8^−10^1^ copies of DHBV DNA and the qPCR cycle conditions were the same as those listed above for DHBV total and cccDNA. In samples where no specific DHBV DNA PCR products were detected, serum DHBV DNA was set at 1/10^th^ of the lower limit of quantitation (LLOQ).

### Statistical analysis

Statistically significant differences in mean body weight, CBE, liver enzymes, mean levels of DHBsAg, DHBV DNA, and anti-DHBs and anti-DHBc antibodies in the serum, and levels of DHBV total and cccDNA in the liver amongst different treatment groups were determined using the Student’s *t* test. Gene expression data are expressed as mean values ± SEM (standard error of mean). Differences between any two groups were determined by Wilcoxon’s test, p<0.05 was considered to be statistically significant.

## Results

### Effect of REP 2055 on induction of cytokines in PHH *in vitro*

**Fig 2:**
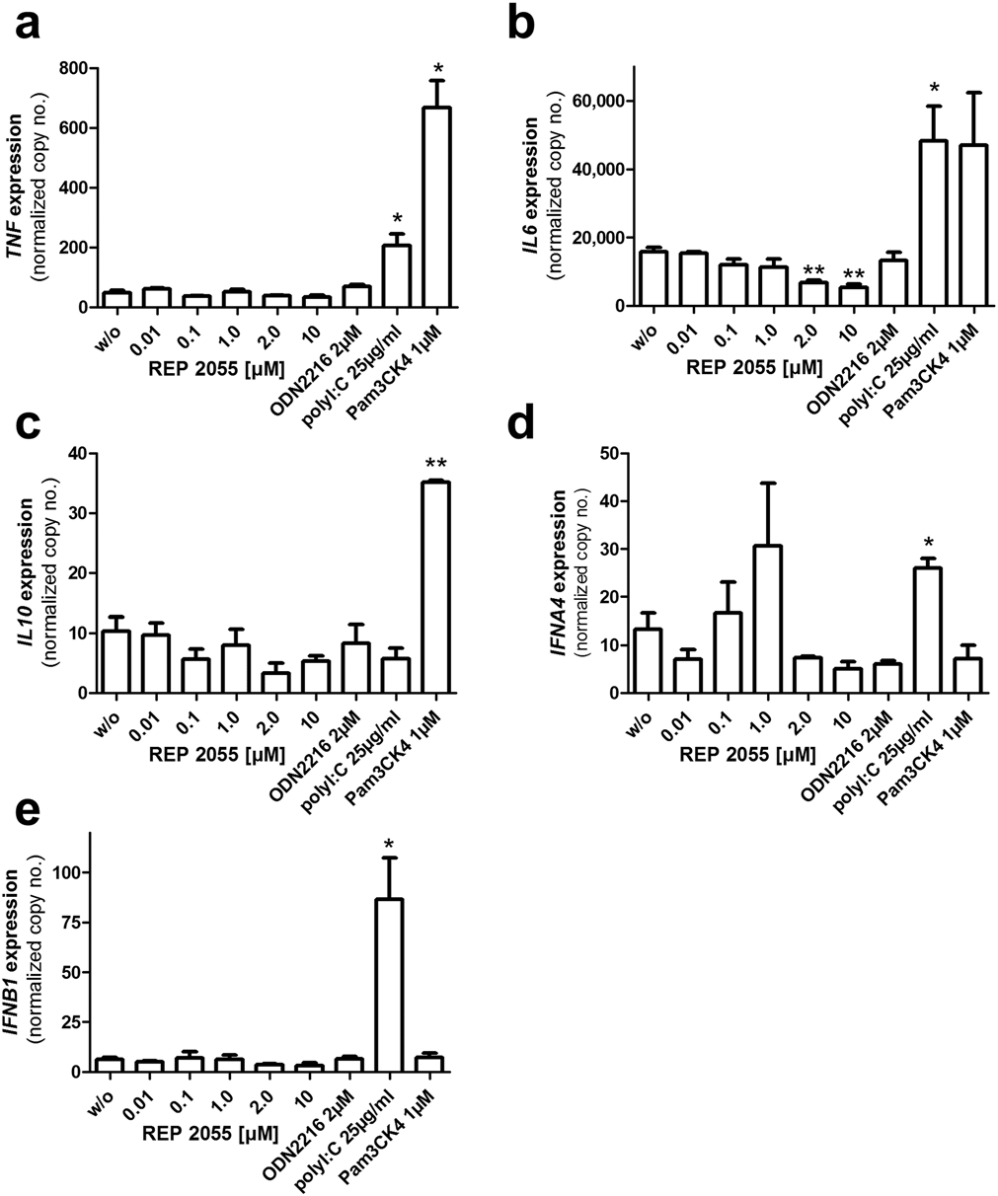
Lack of cytokine gene upregulation in PHH treated with REP 2055. The expression levels of *TNF* (a), *IL6* (b), *IL10* (c), *IFNA4* (d) and *IFNB1* (e) genes were assessed by quantitative RT-PCR 6 hr after treatment with the NAP REP 2055 (0.01–10 μM) or without treatment (w/o), ODN 2216 (a CpG oligonucleotide TLR-9 agonist; 2 μM), poly I:C (a double stranded RNA TLR-3 agonist; 25 μg/ml) or Pam3CK4 (a TLR-1/2 agonist; 1 μM). Values represent mean ± SEM (normalized to 100,000 copies of beta actin mRNA). Statistically significant changes compared to untreated controls are reported for p< 0.05 (*) and p< 0.01 (**).

As assays for duck cytokine gene expression are not currently available in the laboratory, the immunostimulatory properties of REP 2055 were examined in PHH, where cytokine responses involved in antiviral activity are well characterized. As a first step the uptake of REP 2055 by PHH was confirmed as previously described for other NAPs in PDH [16] by treatment of PHH with a CY3-labelled REP 2055 analog followed by examination by fluorescence microscopy (R. Broering, unpublished observation). PHH were then treated with REP 2055 for 6 hr, harvested and assayed by quantitative RT-PCR for the induction of various cytokine genes (Fig 2). Treatment with REP 2055 at concentrations from 0.01–10 μM did not elicit any significant induction of *TNF*, *IL6*, *IL10*, *IFNA4* or *IFNB1* genes whereas all the above genes were induced with control compounds agonizing TLR-1/2 (Pam3CK3) or TLR-3 (poly I:C double stranded RNA). No significant induction of cytokine genes was observed with the TLR-9 agonist ODN 2216 (a single-stranded DNA) consistent with previous reports that TLR-9 functionality may be significantly reduced or absent in PHH [18, 30].

### *In vivo* Experiment 1: Assessing different REP 2055 dosing regimens in DHBV infected ducks

#### Tolerability

Four groups of 5 ducks received different REP 2055 treatment regimens by IP injection as outlined in Fig 1A. All regimens were well tolerated with normal body weight gain during and after treatment (S1 Fig).

#### Antiviral effect

**Fig 3:**
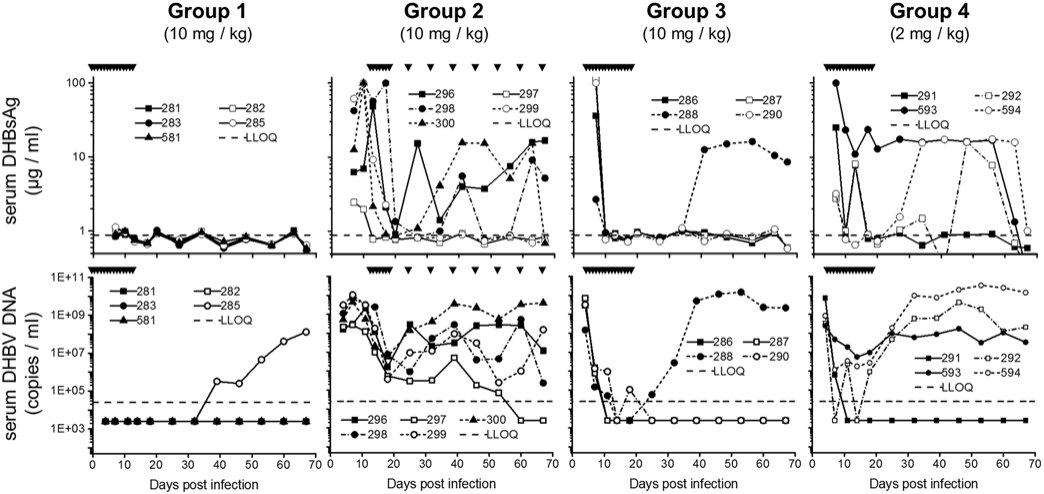
*In vivo* Experiment 1. **Response to various REP 2055 treatment regimens in Groups 1–4**. Individual duck data for serum DHBsAg (top row) and serum DHBV DNA (bottom row). Days of REP 2055 treatment are indicated at the top of each graph by inverted triangles (see also Fig 1A). LLOQ for DHBsAg (0.88 μg/ml) and DHBV DNA (24000 copies/ml) are shown as dashed lines.

In Group 1, REP 2055 (10 mg/kg/day) was used in prophylaxis against DHBV infection (Fig 1A). No evidence of DHBV infection was detected in the serum or liver of 5/5 ducks during treatment and in 4/5 ducks after treatment withdrawal (Table 1, Fig 3, S2 Fig, S3A Fig). In the 1/5 ducks (duck 285) serum DHBV DNA rebounded off treatment and autopsy liver tissue was DHBV DNA, DHBsAg and DHBcAg positive (Table 1, S2B Fig, S3A Fig).

**Table 1:** *In vivo* Experiment 1: Detection of DHBsAg and DHBcAg positive hepatocytes in liver tissue.

			Antigen positive hepatocytes (%)							
			Biopsy Liver Tissue						Autopsy Liver Tissue	
			14 dpi(End of treatment)		12 dpi(Pre-treatment)		18 dpi(End of treatment)		End of Follow-up(Groups 1, 3 and 4)End of treatment (Group 2)	
Treatment Group	REP 2055 treatment regimen	Duck Number	DHBsAg	DHBcAg	DHBsAg	DHBcAg	DHBsAg	DHBcAg	DHBsAg	DHBcAg
1	10 mg/kg IP daily from 1 day prior to infection until 14 dpi	281	<0.001	<0.006	---^a^	---	---	---	<0.001^b^	<0.006^c^
		282	<0.001	<0.006	---	---	---	---	<0.001	<0.006
		283	0.015	<0.006	---	---	---	---	<0.001	<0.006
		285	<0.001	<0.006	---	---	---	---	>95	>95
		581	<0.001	<0.006	---	---	---	---	<0.001	<0.006
2	10 mg/kg IP daily from 12–19 dpi, followed by 7 weekly doses	296	---	---	>95	>95	---	---	>95^d^	>95^d^
		297	---	---	>95	>95	---	---	<0.001	ND^e^
		298	---	---	>95	>95	---	---	>95	>95
		299	---	---	>95	>95	---	---	65.8	76.4
		300	---	---	>95	>95	---	---	>95	ND
3	10 mg/kg IP daily from 4–18 dpi	286	---	---	---	---	82.2	85.2	<0.001	0.006
		287	---	---	---	---	<0.001	<0.006	<0.001	<0.006
		288	---	---	---	---	80.7	82	>95	>95
		290	---	---	---	---	79.9	85	<0.001	<0.006
4	2 mg/kg IP daily from 4–18 dpi	291	---	---	---	---	0.002	<0.006	<0.001	<0.006
		292	---	---	---	---	91.3	94.8	>95	>95
		593	---	---	---	---	95	95	>95	>95
		594	---	---	---	---	90.7	87.2	>95	>95

^a^--- = no liver tissue harvested

^b^Lower limit of detection of DHBsAg positive hepatocytes is 0.001%.

^c^Lower limit of detection of DHBcAg positive hepatocytes is 0.006%.

^d^The liver of duck 296 contained amyloid deposits

^e^ND = not done due to sample exhaustion

In Group 2, ducks with previously established and widespread DHBV infection, received REP 2055 (10 mg/kg/day) (Fig 1A). High levels of DHBV were present in the serum and liver of all ducks prior to the start of treatment at 12 dpi (Table 1, Fig 3, S3B Fig). Daily treatment with REP 2055 for 7 days led to rapid declines in serum DHBsAg (~2 logs) and DHBV DNA (3–5 logs) in all ducks. However, serum DHBsAg and DHBV DNA rebounded in 4/5 ducks upon transition to once weekly dosing (Fig 3). In 1/5 ducks (duck 297), DHBV infection did not rebound and remained supressed, with no evidence of DHBV infection in serum or liver at 68 dpi at the end of treatment (Table 1, Fig 3, S2B Fig, S3B Fig).

Groups 3 and 4 assessed the effects of 14 days of treatment with REP 2055 from 4–18 dpi (Fig 1A). Serum DHBsAg and DHBV DNA were present in all ducks prior to treatment (Fig 3). In the Group 3 ducks, REP 2055 treatment (10 mg/kg/day) reduced serum DHBsAg to below the LLOQ and reduced serum DHBV DNA levels by 5–6 logs in all ducks (Fig 3). In the end of treatment biopsies at 18 dpi, liver DHBV DNA was reduced in all ducks (S2A Fig). During follow-up, 3/4 Group 3 ducks achieved a sustained virological response (SVR): serum DHBsAg and DHBV DNA remained undetectable (Fig 3) and at the end of follow-up at 68 dpi liver tissue had no detectable DHBV DNA, DHBsAg or DHBcAg (Table 1, S2B Fig, S4A Fig). In 1/4 ducks (duck 288), serum DHBsAg and DHBV DNA rebounded off treatment and at the end of follow-up at 68 dpi liver tissue contained high levels of DHBV DNA, DHBsAg and DHBcAg (Table 1, Fig 3, S2B Fig, S4A Fig).

In the Group 4 ducks, although treatment with 2 mg/kg/day of REP 2055 from 4–18 dpi reduced levels of serum DHBsAg to < LLOQ in 3/4 ducks (Fig 3) and led to rapid decreases in serum DHBV DNA of >3 logs in all ducks (Fig 3), serum DHBsAg and DHBV DNA rapidly rebounded in 3/4 ducks (Fig 3). At the end of follow-up at 68 dpi liver tissue contained high levels of DHBV DNA, DHBsAg and DHBcAg (Table 1, S2B Fig, S4B Fig). The remaining duck in this Group (duck 291) achieved an SVR and had no evidence of DHBV infection in the serum or liver at the end of follow-up (Table 1, Fig 3, S2B Fig, S4B Fig).

### *In vivo* Experiment 2: Effect of 28 days of REP 2055 treatment against persistent DHBV infection

#### Tolerability

Ducks with persistent DHBV infection were treated by IP injection with NS (n = 14) or REP 2055 (n = 14) and followed after treatment as described in Fig 1B. During the last 2 weeks of treatment, in 3 ducks in the REP 2055 Group and 1 duck in the NS Group, bacterial infection and moderate to severe inflammation at the injection site necessitating antibiotic treatment developed. These 4 ducks were euthanized due to significant body weight loss and progressive lethargy and autopsy of these ducks revealed peritoneal abscesses likely caused by repeated IP injections. These 4 euthanized ducks were excluded from the experimental data presented below. Overall, a mild but significant reduction in body weight gain, which rapidly normalized during the follow-up, was observed during the last 2 weeks of treatment in the REP 2055 Group (n = 11) compared to the NS Group (n = 13, S5A Fig). During this time a few ducks appeared to faint during the REP 2055 injections with rapid recovery afterward. No incidents of fainting were observed with NS injections.

Local inflammation and associated vascularization is also common at the injection site of PS-ONs in humans [31]. PS-ONs have also been shown to transiently reduce arterial blood pressure when given by bolus intravenous (IV) injection [32]. In the current study development of inflammation and vascularization at the injection site over the 28 days of treatment may have resulted in inadvertent intravenous IV access of REP 2055 that led to the fainting observed during the last 2 weeks of treatment.

Mild decreases in packed red blood cell (RBC) volume and increases in white blood cell (WBC) count occurred during treatment with REP 2055 (S5B Fig, S5C Fig). No changes in levels of the liver enzymes ALT and AST were observed during treatment with REP 2055 or NS but elevations in GGT were observed (S5D Fig, S5E Fig, S5F Fig). No gross pathological changes of internal organs were observed at autopsy (data not shown). Liver tissue from ducks in both treatment groups displayed a hydropic-like vacuolation of hepatocytes that was most prominent at 16 weeks of follow-up (S8A Fig, asterisks) but was absent in the REP 2055-treated ducks that achieved an SVR after treatment withdrawal. Hydropic-like vacuolation of hepatocytes has also been reported in chronically HBV-infected tree shrews [33] suggesting that it is a general consequence of hepadnaviral infection. The absence of hydropic vacuolation in the ducks that achieved an SVR is consistent with the sustained control of DHBV infection observed in these ducks.

#### Antiviral response to 28 days of treatment with 10 mg/kg/day REP 2055

**Fig 4:**
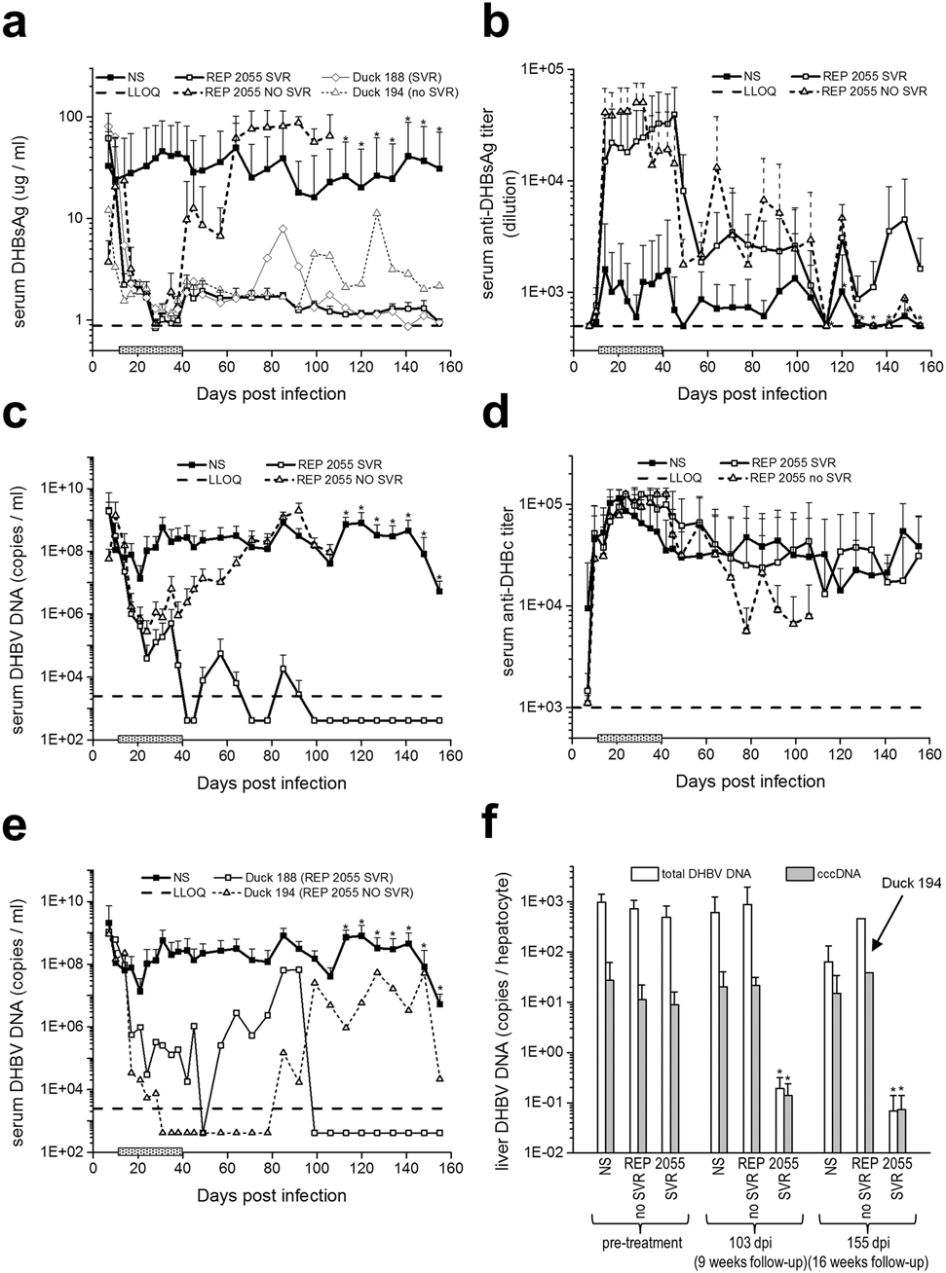
*In vivo* Experiment 2. Response to treatment with NS or REP 2055. Levels of DHBsAg (a), anti-DHBs antibodies (b), DHBV DNA (c and e) and anti-DHBc antibodies (d) in the serum and DHBV total and cccDNA in the liver (f) during and after treatment are shown. For NS-treated ducks in all panels, n = 13 except for the last 7 measurements, where n = 7 (see *). Horizontal lines in (a-e) represent the lower limit of quantification. For REP 2055duck n = 6 but SVR duck 188 is plotted separately in (a) and (e). For REP 2055 NO SVR ducks, n = 5 but NO SVR duck 194 is plotted separately in (a) and (e). Duck 194 and duck 188 are plotted separately in (a) and (e) as the response of these ducks was different from the other SVR or NO SVR ducks. Treatment interval in (a-e) is indicated on the x-axis by the shaded bar. Differences in mean responses of REP 2055 SVR and REP 2055 NO SVR were significantly different from NS in a, b and c (p< 0.05). Differences in mean responses of REP 2055 SVR were not significantly different from REP 2055 NO SVR in (b). In (e), ** = statistically different from NS or REP 2055 NO SVR groups (p < 0.01).

High levels of DHBV infection were present in the serum and liver of all ducks before treatment with NS or REP 2055 (Table 2, Fig 4, S6 Fig). Anti-DHBc antibodies developed rapidly in all ducks following DHBV infection and were not affected by NS or REP 2055 treatment and persisted throughout follow-up (Fig 4D). In the NS Group (n = 13), DHBV infection was unaffected by treatment and high levels of DHBV infection were present in serum and liver during treatment and follow-up (Table 2, Fig 4, S7A Fig, S8A Fig).

**Table 2:** *In vivo* Experiment 2: Detection of DHBsAg and DHBcAg positive hepatocytes in liver tissue.

Treatment^a^(12–40 dpi)	Duck Number	Antigen positive hepatocytes (%)					
		Biopsy 1(pre-treatment, 12 dpi)		Biopsy 2 / Autopsy(103 dpi, 9 weeks follow-up)		Autopsy(155 dpi, 16 weeks follow-up)	
		DHBsAg	DHBcAg	DHBsAg	DHBcAg	DHBsAg	DHBcAg
NS(1 ml/kg/day)	170	>95	>95	>95	89.9	>95	>95
	172	>95	>95	>95	>95	---^b^	---
	174	>95	>95	>95	>95	---	---
	176	>95	>95	>95	81.3	>95	>95
	177	>95	>95	>95	>95	---	---
	178	>95	>95	>95	90.4	>95	>95
	180	>95	>95	>95	88.7	>95	>95
	181	>95	>95	>95	>95	>95	>95
	182	>95	>95	>95	>95	>95	>95
	183	>95	>95	>95	>95	---	---
	185	>95	>95	>95	>95	>95	>95
	186	>95	>95	>95	>95	---	---
	193	>95	>95	23.6	23.4	---	---
REP 2055(10 mg/kg/day)	179	>95	>95	<0.001^c^	<0.006^d^	<0.001	<0.006
	187	>95	>95	<0.001	<0.006	<0.001	<0.006
	188	>95	>95	<0.001	<0.006	<0.001	<0.006
	189	>95	>95	>95	>95	---	---
	191	>95	>95	>95	>95	---	---
	192	>95	>95	>95	>95	---	---
	194	>95	>95	>95	86.8	>95	> 95
	196	>95	>95	<0.001	<0.006	<0.001	<0.006
	197	>95	>95	<0.001	<0.006	<0.001	<0.006
	198	>95	>95	<0.001	<0.006	<0.001	<0.006
	199	>95	>95	>95	>95	---	---

^a^NS or REP 2055 was administered via IP injection.

^b^--- = no liver tissue harvested (ducks autopsied at 103 dpi, 9 weeks of follow-up)

^c^Lower limit of detection of DHBsAg positive hepatocytes is 0.001%.

^d^Lower limit of detection of DHBcAg positive hepatocytes is 0.006%.

All ducks treated with REP 2055 (n = 11) experienced initial rapid reductions in serum DHBsAg (~2 logs) and serum DHBV DNA (~3 logs) and increases in serum anti-DHBs antibody titers by the end of the 2^nd^ week of treatment (Fig 4). REP 2055 treatment led to the emergence of 2 groups of ducks; those that rebounded during follow-up (NO SVR; n = 5) and those that achieved an SVR at the end of follow-up (n = 6). Data from the REP 2055 NO SVR and SVR ducks are grouped separately for the results presented below.

In the REP 2055 NO SVR ducks (n = 5), no further reductions in serum DHBV DNA beyond the initial ~ 3 log drop were observed during the 3^rd^ and 4^th^ weeks of treatment. During follow-up, serum DHBV DNA and DHBsAg rapidly rebounded within 1–4 weeks to pre-treatment levels (Fig 4A and 4C) and serum anti-DHBs antibody titers declined but were still greater than in the NS-treated ducks (Fig 4B). In 1 duck (duck 194), the rebound in serum DHBsAg and DHBV DNA was delayed compared to the other NO SVR ducks (Presented separately in Fig 4E). At 9 and 16 weeks of follow-up, levels of DHBsAg and DHBV DNA in the serum and levels of DHBsAg and DHBcAg and DHBV total and cccDNA in the liver of all 5 NO SVR ducks remained at persistently high levels (Table 2, Fig 4F, S7B Fig).

In the REP 2055 SVR ducks (n = 6), serum DHBsAg and DHBV DNA titers continued to decline during the 3^rd^ and 4^th^ weeks of treatment and became < LLOQ (5–7 log reduction) by the end of treatment (Fig 4C). During follow-up serum DHBsAg and DHBV DNA were undetected (Fig 4A and 4B) and although levels of serum anti-DHBs antibody decreased they were higher than in the NS-treated ducks (Fig 4C). In 1 duck (duck 188), an initial rebound in serum DHBV DNA observed during the first 8 weeks of follow-up was followed by spontaneous reduction to < LLOQ which was maintained for the remainder of the follow-up (Presented separately in Fig 4E). At 9 and 16 weeks of follow-up, no DHBsAg or DHBcAg positive hepatocytes were detected in the liver (Table 2, S7B Fig, S8B Fig) and levels of DHBV total (0.195 ± 0.12 and 0.069 ± 007 copies / hepatocyte) and cccDNA (0.138 ± 0.01 and 0.072 ± 0.067 copies / hepatocyte) were reduced. These REP 2055-mediated reductions in DHBV total and cccDNA at 16 weeks of follow-up represent reductions of 928-fold and 205-fold respectively from levels present in the livers of NS-treated ducks at 16 weeks of follow-up and are consistent with the establishment of sustained control of DHBV infection.

## Discussion

Although other members of the chemical class to which NAPs belong (PS-ONs) are known to have immunostimulatory activity due to the presence of CpG motifs, it has been shown in other virus systems [13] that the immunostimulatory activity of NAPs can be eliminated by altering their sequence to exclude CpG motifs without affecting their antiviral activity *in vivo*. Similar findings have been demonstrated in DHBV infection by designing NAPs without CpG motifs then testing for immunostimulation and antiviral activity *in vivo*: REP 2031 (a phosphorothioated 40 mer NAP whose amphipathic activity is selectively neutralized at acidic pH) lacked antiviral activity while REP 2055 (a phosphorothioated 40 mer NAP that is not inactivated at acid pH) was highly active against DHBV infection. However, with the absence of CpG motifs neither NAP caused direct immunostimulation [16, 17]. In contrast REP 2006, which by virtue of its degenerate nature includes CpG motifs, showed classic proinflammatory side effects and was immunostimuatory in DHBV infected ducks [17].

Additional data presented here shows that REP 2055 does not activate cytokine gene expression in PHH (Fig 2), also suggesting that the antiviral effect of REP 2055 *in vivo* is not caused by a direct immunostimulatory effect. Notwithstanding these observations, the restoration of immunological function observed in these studies that results in the sustained control of DHBV infection warrants the continued investigation of possible direct immunostimulatory effects of NAPs against DHBV and HBV infection.

Experiment 1, which was designed to assess different REP 2055 dosing regimens, confirmed in the Group 1 ducks the previously reported prophylactic activity of REP 2055 against DHBV infection [17]. Experiment 1 also demonstrated that treatment with REP 2055 was able to reduce levels of DHBV infection in the serum and liver of ducks with previously established DHBV infection: Treatment with REP 2055 at 10 mg/kg/day for 7 days (Group 2) reduced levels of DHBV, however, transition from daily to weekly treatment led to viral rebound, demonstrating that once weekly dosing could not maintain therapeutically active levels of REP 2055. Extending daily treatment to 14 days (Group 3) reduced levels of DHBV in the serum and ultimately resulted in an SVR in 3/4 ducks. 14 days of treatment with 2 mg/kg/day (Group 4) reduced levels of DHBV but only achieved an SVR in 1/4 ducks.

Although NAPs have antiviral effects during and after DHBV viral entry *in vitro*, only the post-entry antiviral activity of NAPs was shown to produce an antiviral effect *in vivo* [17]. Thus the antiviral effects of REP 2055 in Experiment 1 demonstrate that the post-entry antiviral activity of REP 2055 can be effective not only in prophylaxis but also to treat pre-established DHBV infection.

A larger *in vivo* experiment, Experiment 2, was designed to include treatment of ducks with REP 2055 at 10 mg/kg/day for 28 days. In this experiment 14-day-old ducks were infected with DHBV and then treated with REP 2055 starting at 12 dpi where >95% of hepatocytes were infected with DHBV. All REP 2055 treated ducks experienced initial rapid reductions in serum DHBsAg and serum DHBV DNA and increases in serum anti-DHBs antibody titers during the 1^st^ and 2^nd^ weeks of treatment. As explained above REP 2055 treatment led to the emergence of 2 groups of ducks, with and without SVR. In the 6/11 REP 2055 SVR ducks, serum DHBsAg and DHBV DNA titers continued to decline and became undetectable by the end of treatment. After REP 2055 treatment was withdrawn serum DHBsAg and DHBV DNA remained undetected but anti-DHBs antibodies persisted. In the liver, no DHBsAg or DHBcAg antigen positive hepatocytes and only trace amounts of DHBV total and cccDNA were detected at the end of 9 or 16 weeks of follow-up.

The absence of DHBsAg and DHBcAg positive hepatocytes and detection of only trace amounts of DHBV total and cccDNA in the SVR ducks is reminiscent of the resolution of acute DHBV infection in adult ducks [24, 27, 29], woodchuck hepatitis virus (WHV) infected adult woodchucks [34] and HBV infected chimpanzees [35, 36] where DHBV-, WHV- and HBV-infected hepatocytes are targeted and are cleared from the liver by the immune response.

We hypothesize that the reduction in cccDNA copy number observed in the SVR ducks is caused by renewed or augmented cell mediated immune responses in the liver [35, 36] and suggests recovery of the adaptive immune response. The innate immune response has also been implicated in the non-cytotoxic loss of HBV replicative intermediates and transcriptional inactivation of cccDNA [37, 38]. The transcriptional inactivation and reduction in DHBV infected hepatocytes and DHBV total and cccDNA in Experiment 2 (Fig 4F) were perfectly correlated with SVR, suggesting that both adaptive and innate immune function may be essential for the sustained control of DHBV infection.

It should be noted that rapid gain in body weight during REP 2055 treatment (3-fold increase in body weight from 12–40 dpi; S5A Fig) is accompanied by significant mitosis in the growing liver [28] which may have played a role in the observed antiviral efficacy of REP 2055.

The low levels of DHBV DNA detected in the REP 2055 treated SVR ducks, particularly at 16 weeks of follow-up, indicate that the DHBV infections were well controlled and that infection did not rebound from the trace amounts of cccDNA remaining in the liver. It is interesting to note that at follow-up the ratio of DHBV total to cccDNA was approximately 1 implying that transcription of cccDNA and subsequent production of replicative intermediates was silenced in these ducks. Again this is reminiscent of the resolution of acute DHBV infection in adult ducks where trace amounts of residual DHBV DNA persist without reactivation [24, 27, 29].

In contrast, DHBV infection has been shown to rebound within 30 days of withdrawal of ETV [39] and within 7 days of withdrawal of penciclovir and adefovir [40, 41] suggesting that REP 2055 treatment is more effective at achieving sustained control of DHBV infection than virus polymerase inhibitors in the DHBV model [39–41].

In Experiment 1, REP 2055 suppressed serum DHBsAg in Group 3 and 4 but many hepatocytes remained DHBsAg and DHBcAg positive at the end of treatment (Fig 2, S4 Fig), suggesting that REP 2055 does not directly interfere with viral protein synthesis in infected hepatocytes but instead may block the release of DHBsAg particles. Additionally, 2/4 Group 4 ducks (292 and 594) from Experiment 1 and 4/5 NO SVR ducks and 1/6 SVR ducks from Experiment 2 had detectable levels of serum DHBV DNA (10^5^–10^6^ copies/ml), despite the disappearance of serum DHBsAg (Figs 2, 4A, 4C and 4E), suggesting that REP 2055 may not be able to completely block the release of virions. In HBV as well as DHBV infection, subviral particles (SVPs) constitute more than 99.99% of circulating surface antigen [42, 43] and the unusual observation of suppression of serum DHBsAg but not DHBV DNA with REP 2055 may be due to differences in the sensitivity of the ELISA and PCR assays but is also consistent with a selective inhibition of the release of DHBV SVP. Additional experiments are underway to determine if NAPs have a differential effect on secretion of HBV surface antigen (HBsAg) SVP and HBV virions.

The ability of REP 2055 treatment to elicit SVR in DHBV infected ducks may be derived from the unique effect of REP 2055 to lower DHBsAg levels in the bloodstream during treatment; other antiviral agents that do not block release of DHBsAg have a limited ability to lower DHBsAg in the bloodstream or to achieve SVR in DHBV-infected ducks [39–41]. Lowering DHBsAg in the bloodstream may be an important driver in the control of DHBV infection: HBsAg has been shown to directly inhibit both innate and adaptive immune function [44–49] in peripheral and liver immunity, functions which may be also conserved in DHBsAg. Therefore, it is important to consider the possibility that the restored immune function observed in NAP-treated DHBV-infected ducks could be a result of the removal of DHBsAg (and its accompanying immunoinhibitory properties) from the circulation. As an aside, it is also recognized that the disappearance of HBsAg from the bloodstream in patients receiving antiviral treatment for CHB is a highly reliable predictor for control of HBV infection being maintained after treatment withdrawal [50, 51].

In Experiment 2, co-detection of DHBsAg and anti-DHBs antibodies was observed in the NS treated control ducks (Fig 4A and 4B), consistent with previous reports in ducks with persistent DHBV infection [21–23, 39]. The co-detection of DHBsAg and anti-DHBs antibodies has been proposed to reflect a balance between virus infection and the immune response, with fluctuating levels of DHBsAg and anti-DHBs antibodies, forming immune complexes and that are removed from the bloodstream. However, several studies have demonstrated the presence of immune escape surface antigen in DHBV and HBV infection [52–56] suggesting that the DHBsAg observed in the presence of anti-DHBs antibodies may also reflect DHBsAg which is non-immunoreactive and is unable to be bound and cleared from the bloodstream.

In all ducks REP 2055 treatment led to a reduction of serum DHBsAg and an increase in anti-DHBs antibodies. Following treatment 6/11 REP 2055 treated ducks achieved an SVR. However, in the remaining 5/11 NO SVR ducks cessation of treatment led to rapid viral rebound (Fig 4F). During treatment the NO SVR ducks maintained significant levels of DHBV DNA (10^5^−10^6^ genomes/ml) despite the presence of elevated levels of anti-DHBs antibodies and reductions in DHBsAg. It is possible that this DHBV DNA indicates the presence DHBV strains encoding DHBsAg that is unable to be bound by anti-DHBs antibodies and therefore is not cleared from the bloodstream. It has not been determined if the presence of DHBV strains encoding non-immunoreactive DHBsAg ultimately leads to the treatment failure.

The antiviral effects of REP 2055 in DHBV-infected ducks are clearly superior to those observed with other antiviral therapies in this model [39–41] and strongly suggest that if NAPs were similarly able to clear serum HBsAg in patients with CHB infection sustained control of CHB infection might be also achieved. Limited proof of concept trials in patients with CHB infection treated by once weekly dosing with REP 2055, a regimen routinely employed for other PS-ONs in clinical development, have replicated the antiviral effects observed in DHBV infection *in vivo* (A. Vaillant, unpublished observation). Additional clinical trials are underway to examine the potential of NAPs as a new therapeutic approach for CHB infection of humans.

## Supporting Information

S1 Fig *In vivo* Experiment 1 total body weight. Plotted values represent average duck body weight +/- SD. For Groups 1 and 2, n = 5, for Groups 3 and 4, n = 4. There was no statistically significant difference in mean body weight between any of the Groups.
(TIF)

S2 Fig *In vivo* Experiment 1 liver DHBV DNA. Southern blot hybridisation detection of DHBV DNA in 2 μg DNA samples extracted from liver tissue from ducks in Groups 1–4 at biopsy (a) and autopsy (b). Size marker is pBL4.8x2 cut with Pvu I and Eco RI to yield fragments of 3027, 1708 and 1044 bp. Autoradiographic exposure, 72 hr.
(TIF)

S3 Fig *In vivo* Experiment 1 Groups 1 and 2 Liver DHBsAg and DHBcAg. Detection of DHBsAg and DHBcAg positive hepatocytes by immunostaining of biopsy and autopsy tissue from ducks in Groups 1 (a) and 2 (b). * Unstained regions are amyloid deposits which developed in the liver of duck 296. Magnification 20x; scale bar = 100 μm.
(TIF)

S4 Fig *In vivo* Experiment 1 Groups 3 and 4 liver DHBsAg and DHBcAg. Detection of DHBsAg and DHBcAg positive hepatocytes by immunostaining of biopsy and autopsy tissue from ducks in Groups 3 (a) and 4 (b). Magnification 20x; scale bar = 100 μm).
(TIF)

S5 Fig *In vivo* Experiment 2 tolerability. Total body weight (a), packed RBC volume (b), WBC count (c), and serum GGT (d), ALT (e) and AST (f) are shown for NS (n = 13) and REP 2055 (n = 11) Groups. Values are average +/- SD. Statistically significant differences between NS and REP 2055 Groups are indicated by p-values in (b-f) and * in (a) (p< 0.05).
(TIF)

S6 Fig *In vivo* Experiment 2 pre-treatment liver DHBsAg and DHBcAg. Detection of DHBsAg and DHBcAg positive hepatocytes by immunostaining of biopsy liver tissue collected prior to treatment of ducks with NS (a) and REP 2055 (b). Magnification 20x; scale bar = 100 μm.
(TIF)

S7 Fig *In vivo* Experiment 2 liver DHBsAg and DHBcAg at 9 weeks of follow-up. Detection of DHBsAg and DHBcAg positive hepatocytes by immunostaining of biopsy and autopsy liver tissue collected at 103 dpi (9 weeks of follow-up) in ducks treated with NS (a) and REP 2055 (b). Magnification 20x; scale bar = 100 μm.
(TIF)

S8 Fig *In vivo* Experiment 2 liver DHBsAg and DHBcAg at 16 weeks of follow-up. Detection of DHBsAg and DHBcAg positive hepatocytes by immunostaining of autopsy liver tissue collected at 155 dpi (16 weeks of follow-up) in ducks treated with NS (a) and REP 2055 (b). Prominent hydropic vacuolation of hepatocytes is visible in fields indicated by an *. Magnification 20x; scale bar = 100 μm.
(TIF)
