# Supplementary figures and images for: Therapeutic Antiviral Effect of the Nucleic Acid Polymer REP 2055 against Persistent Duck Hepatitis B Virus Infection
Source: PLoS One. 2015 Nov 11;10(11):e0140909. doi: 10.1371/journal.pone.0140909 (PMC4641618; doi:10.1371/journal.pone.0140909)

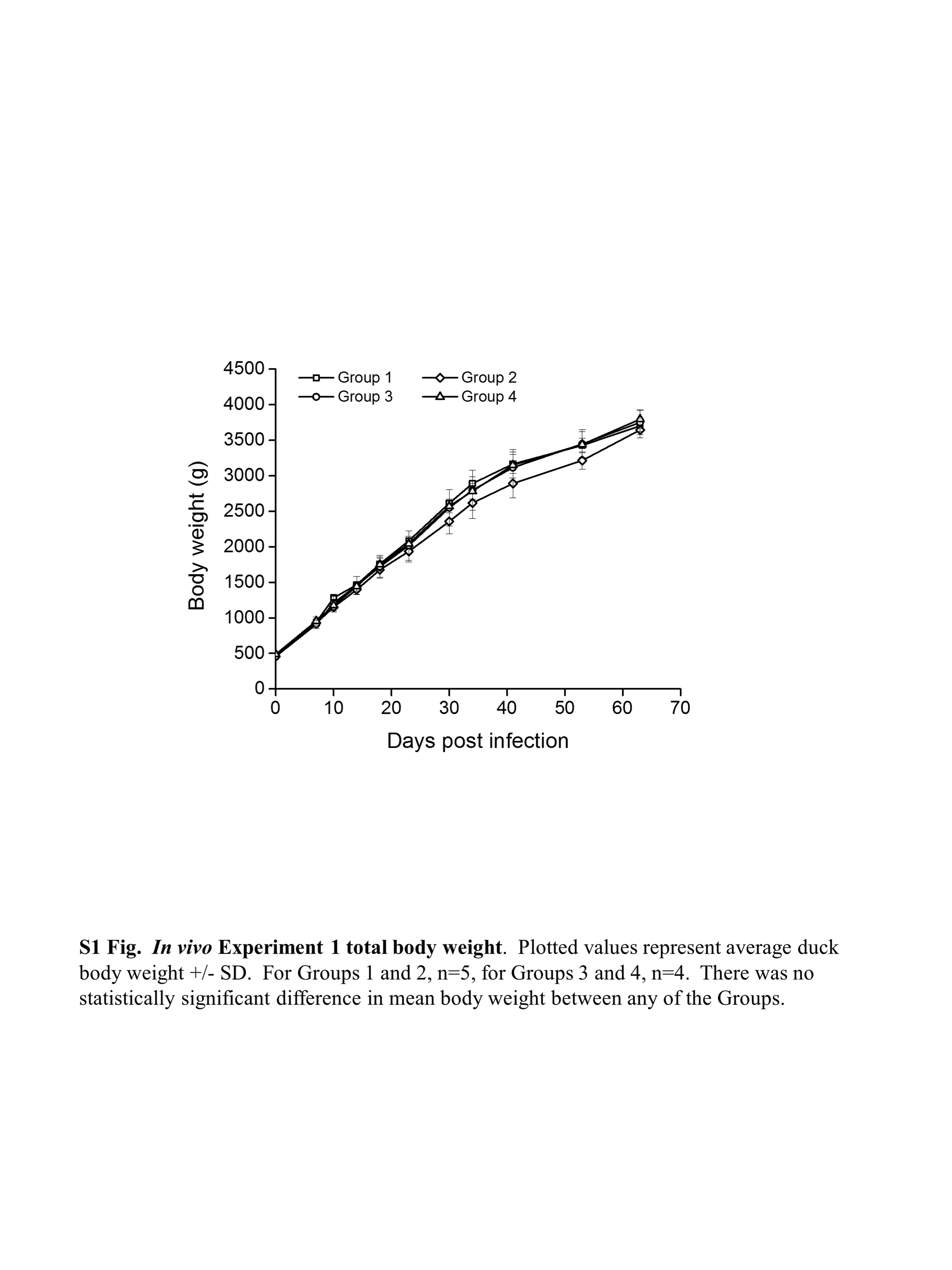

Supplement: S1 Fig — Plotted values represent average duck body weight +/- SD. For Groups 1 and 2, n = 5, for Groups 3 and 4, n = 4. There was no statistically significant difference in mean body weight between any of the Groups. (TIF) [file pone.0140909.s001.tif]

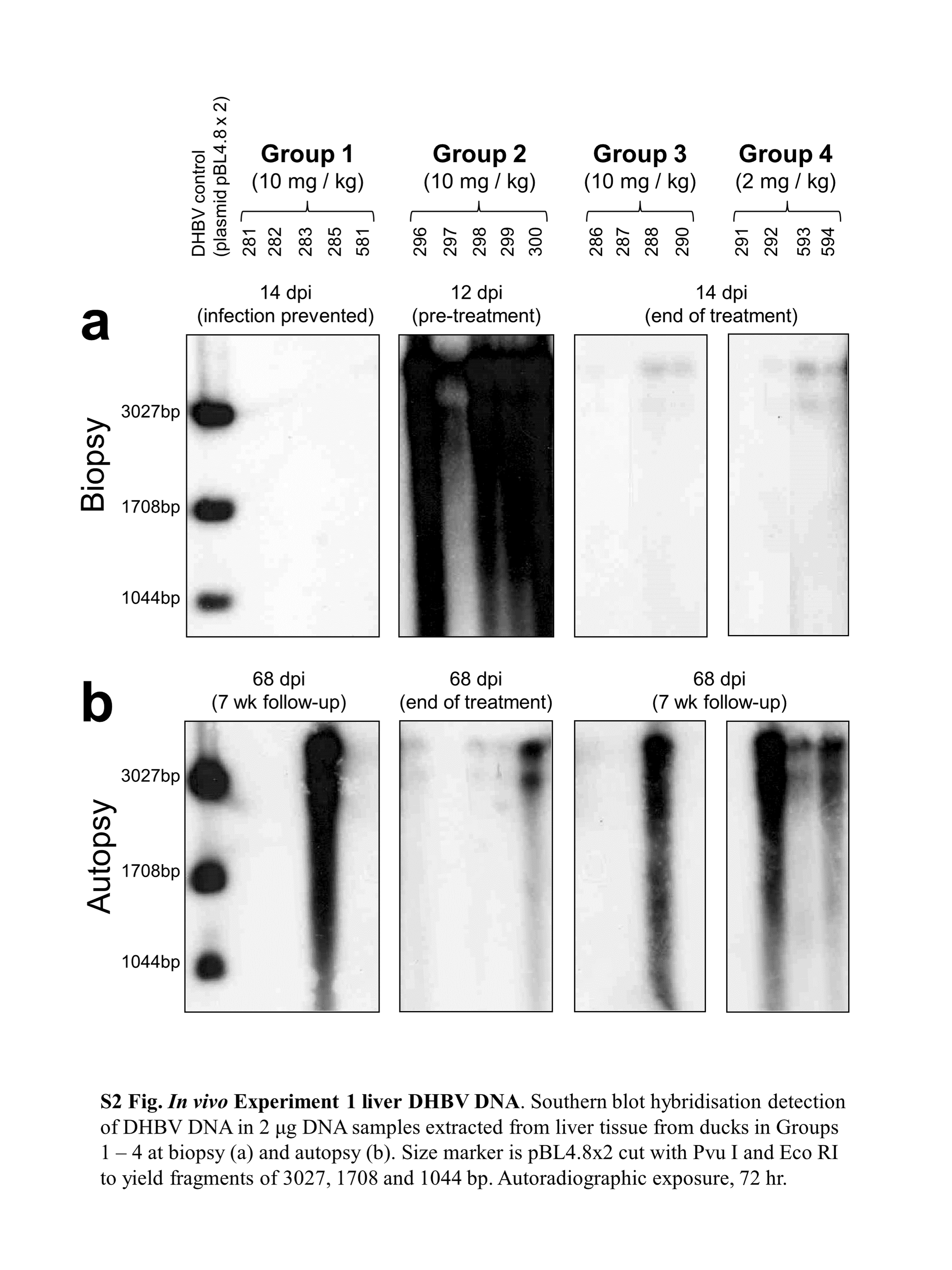

Supplement: S2 Fig — Southern blot hybridisation detection of DHBV DNA in 2 μg DNA samples extracted from liver tissue from ducks in Groups 1–4 at biopsy (a) and autopsy (b). Size marker is pBL4.8x2 cut with Pvu I and Eco RI to yield fragments of 3027, 1708 and 1044 bp. Autoradiographic exposure, 72 hr. (TIF) [file pone.0140909.s002.tif]

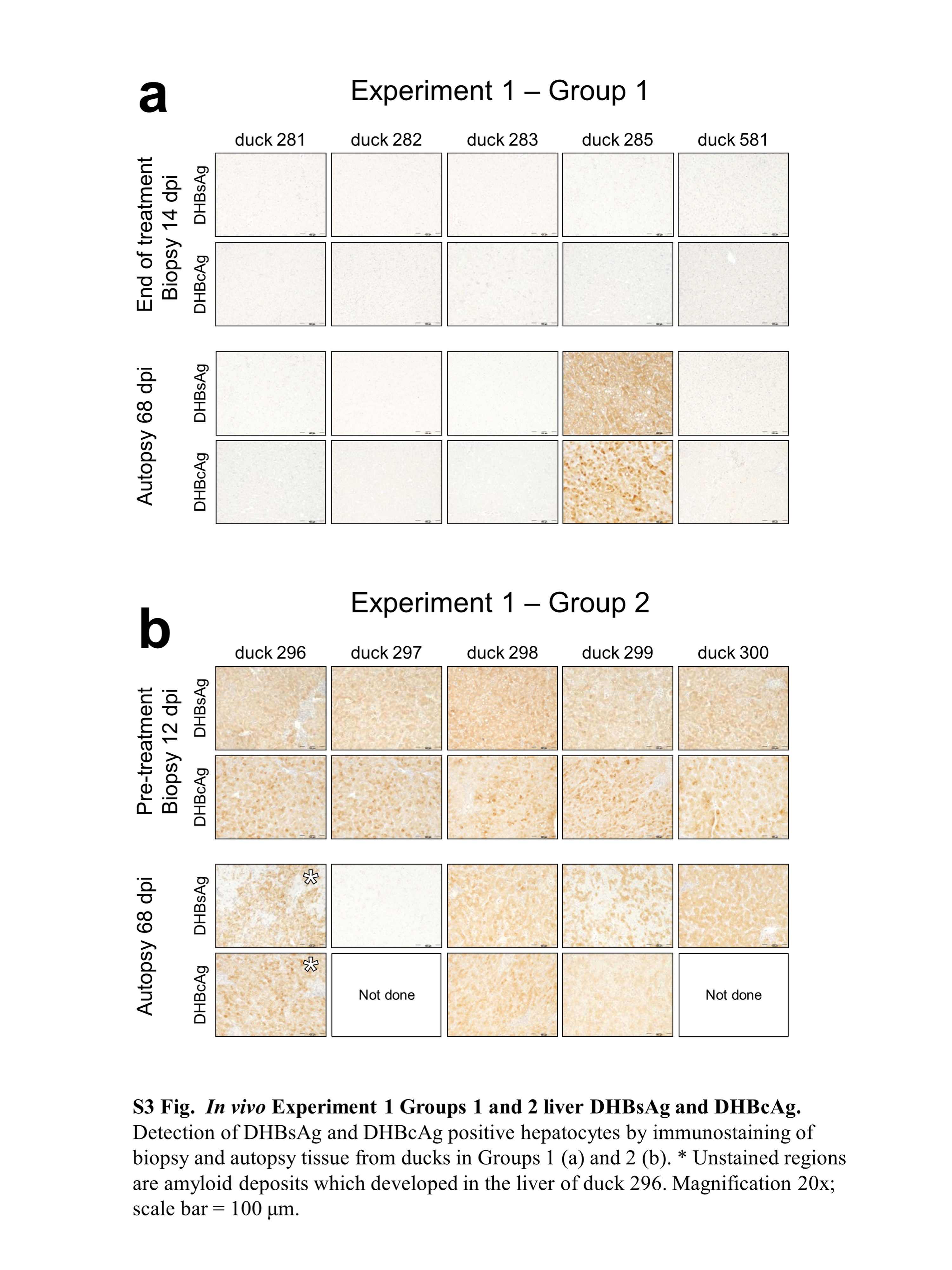

Supplement: S3 Fig — Detection of DHBsAg and DHBcAg positive hepatocytes by immunostaining of biopsy and autopsy tissue from ducks in Groups 1 (a) and 2 (b). * Unstained regions are amyloid deposits which developed in the liver of duck 296. Magnification 20x; scale bar = 100 μm. (TIF) [file pone.0140909.s003.tif]

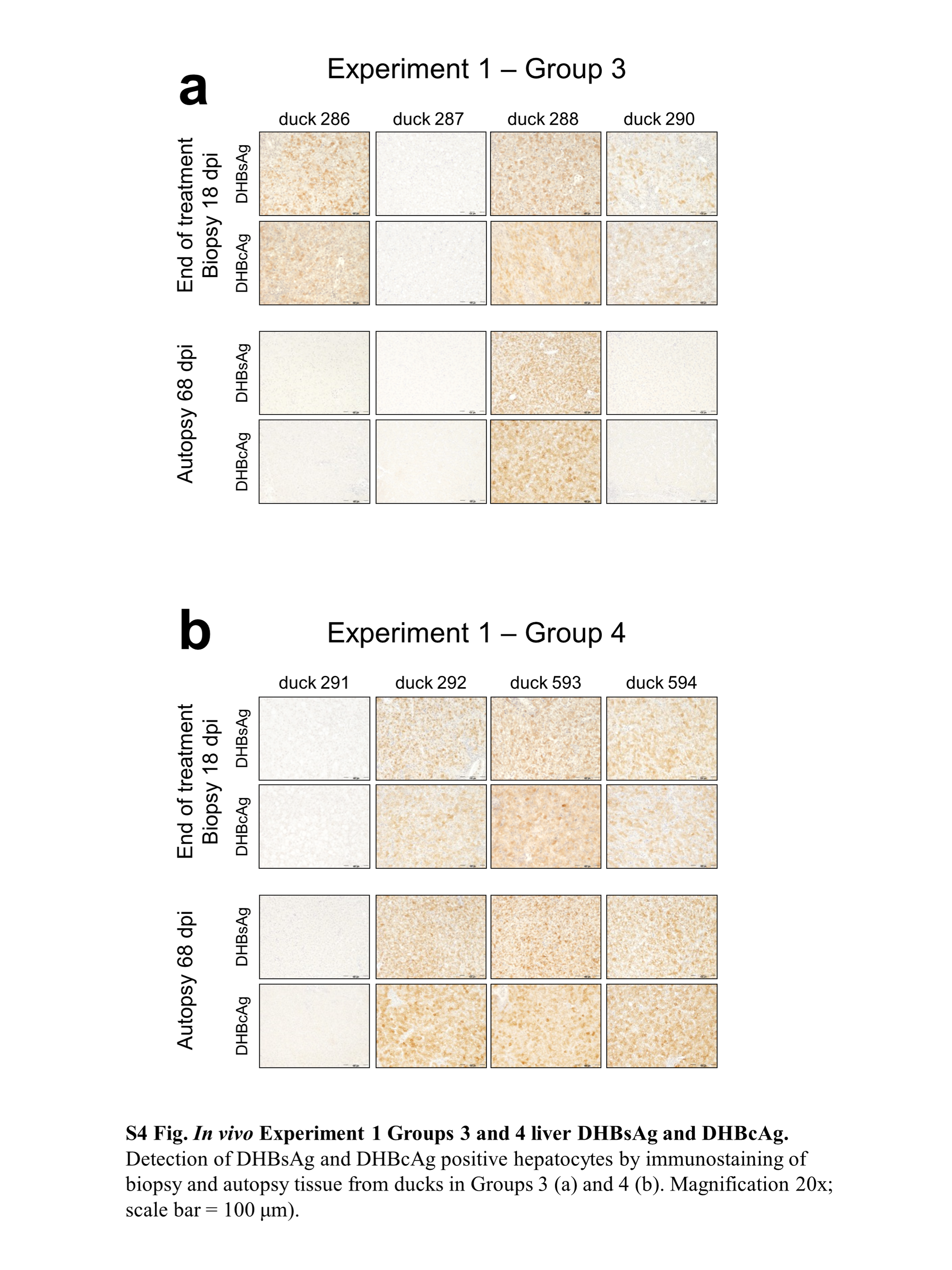

Supplement: S4 Fig — Detection of DHBsAg and DHBcAg positive hepatocytes by immunostaining of biopsy and autopsy tissue from ducks in Groups 3 (a) and 4 (b). Magnification 20x; scale bar = 100 μm). (TIF) [file pone.0140909.s004.tif]

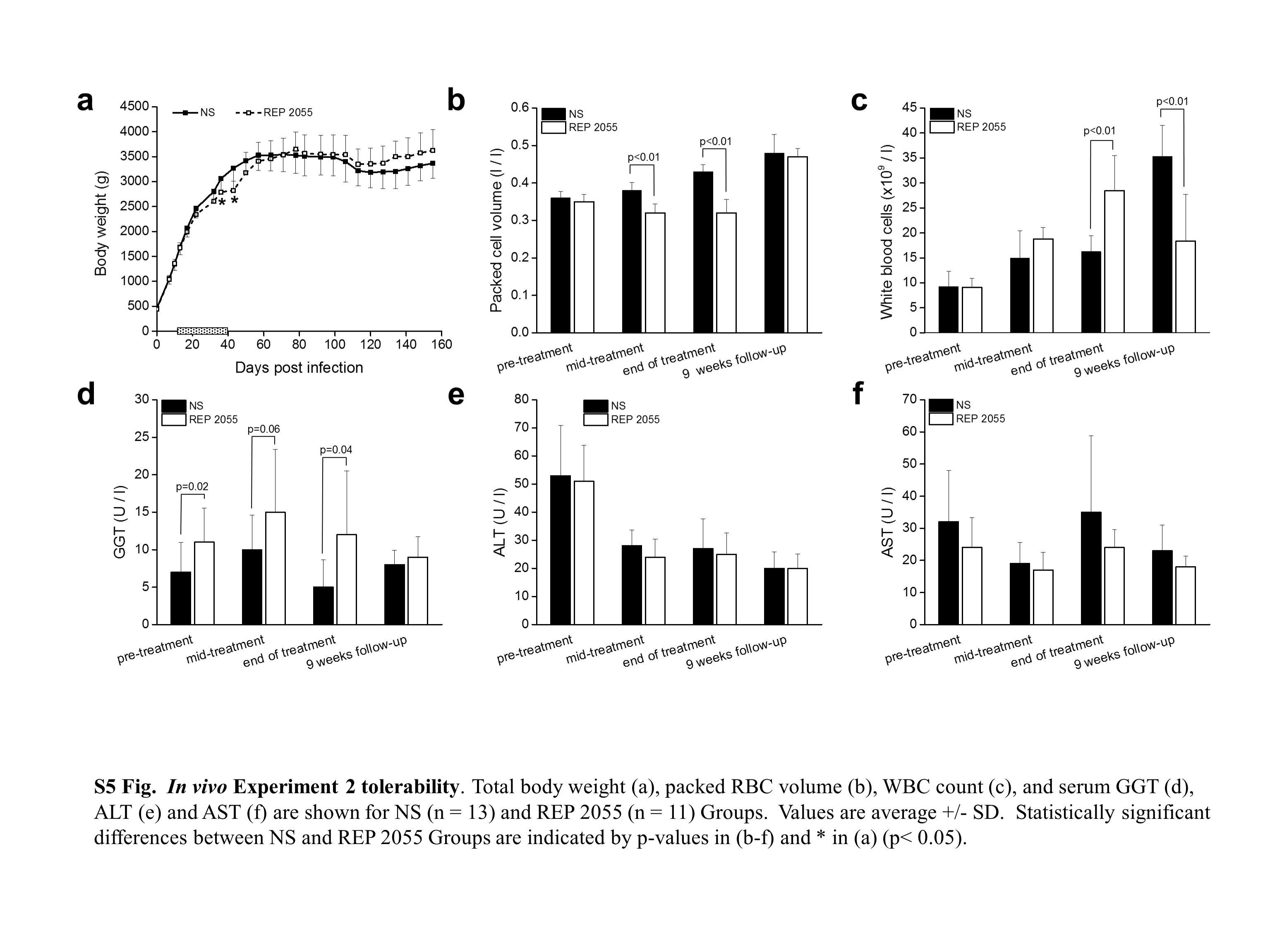

Supplement: S5 Fig — Total body weight (a), packed RBC volume (b), WBC count (c), and serum GGT (d), ALT (e) and AST (f) are shown for NS (n = 13) and REP 2055 (n = 11) Groups. Values are average +/- SD. Statistically significant differences between NS and REP 2055 Groups are indicated by p-values in (b-f) and * in (a) (p< 0.05). (TIF) [file pone.0140909.s005.tif]

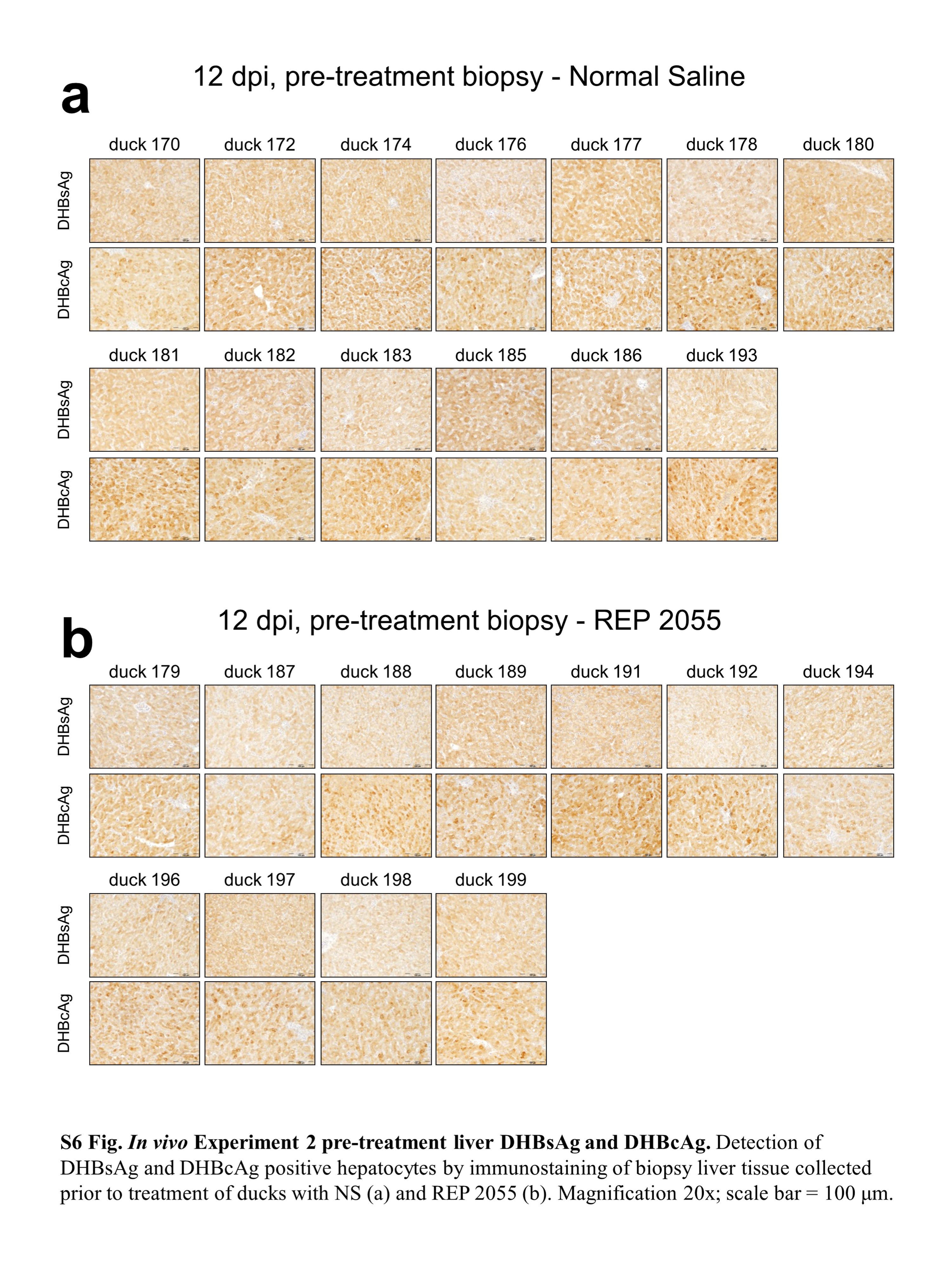

Supplement: S6 Fig — Detection of DHBsAg and DHBcAg positive hepatocytes by immunostaining of biopsy liver tissue collected prior to treatment of ducks with NS (a) and REP 2055 (b). Magnification 20x; scale bar = 100 μm. (TIF) [file pone.0140909.s006.tif]

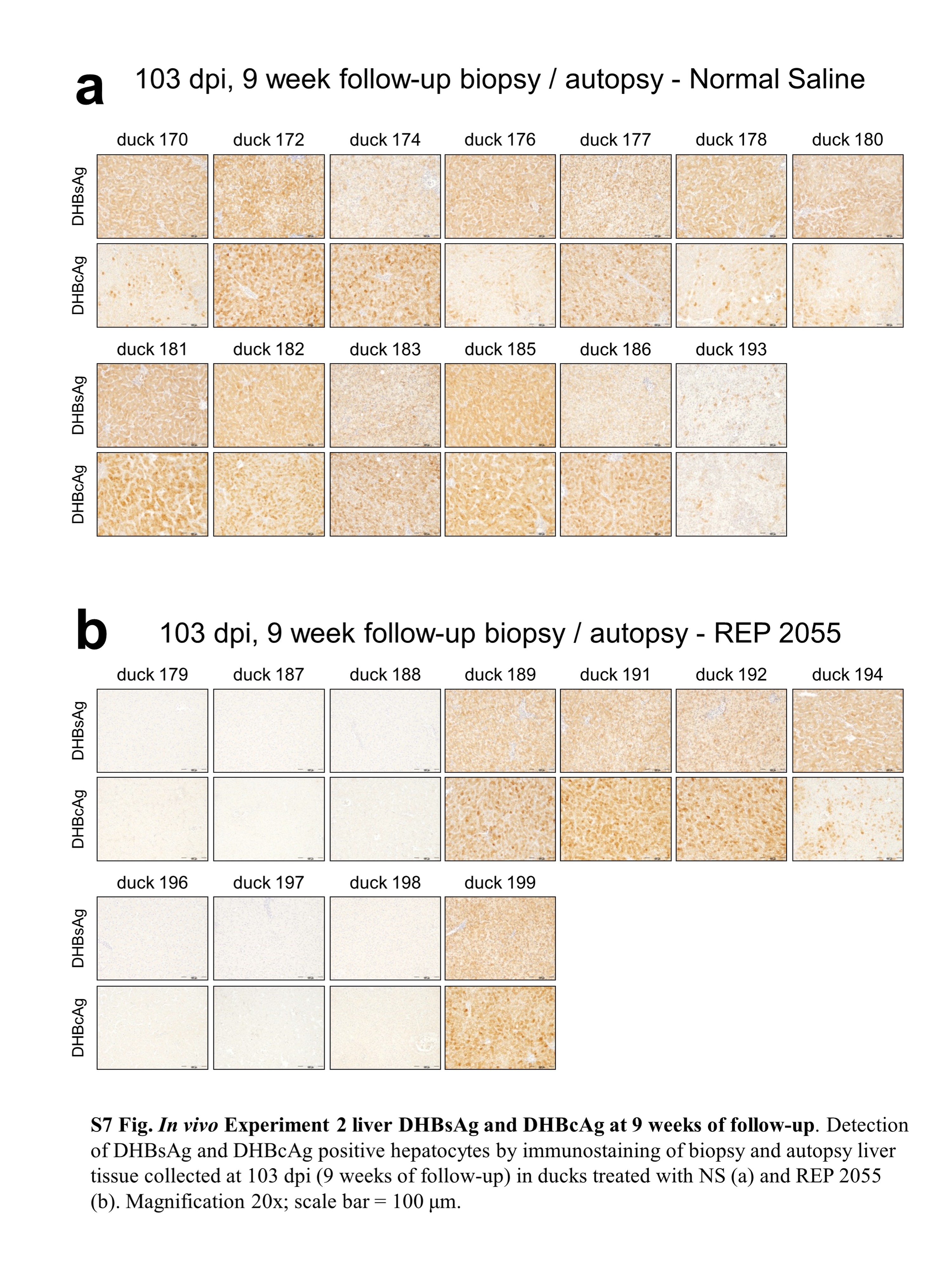

Supplement: S7 Fig — Detection of DHBsAg and DHBcAg positive hepatocytes by immunostaining of biopsy and autopsy liver tissue collected at 103 dpi (9 weeks of follow-up) in ducks treated with NS (a) and REP 2055 (b). Magnification 20x; scale bar = 100 μm. (TIF) [file pone.0140909.s007.tif]

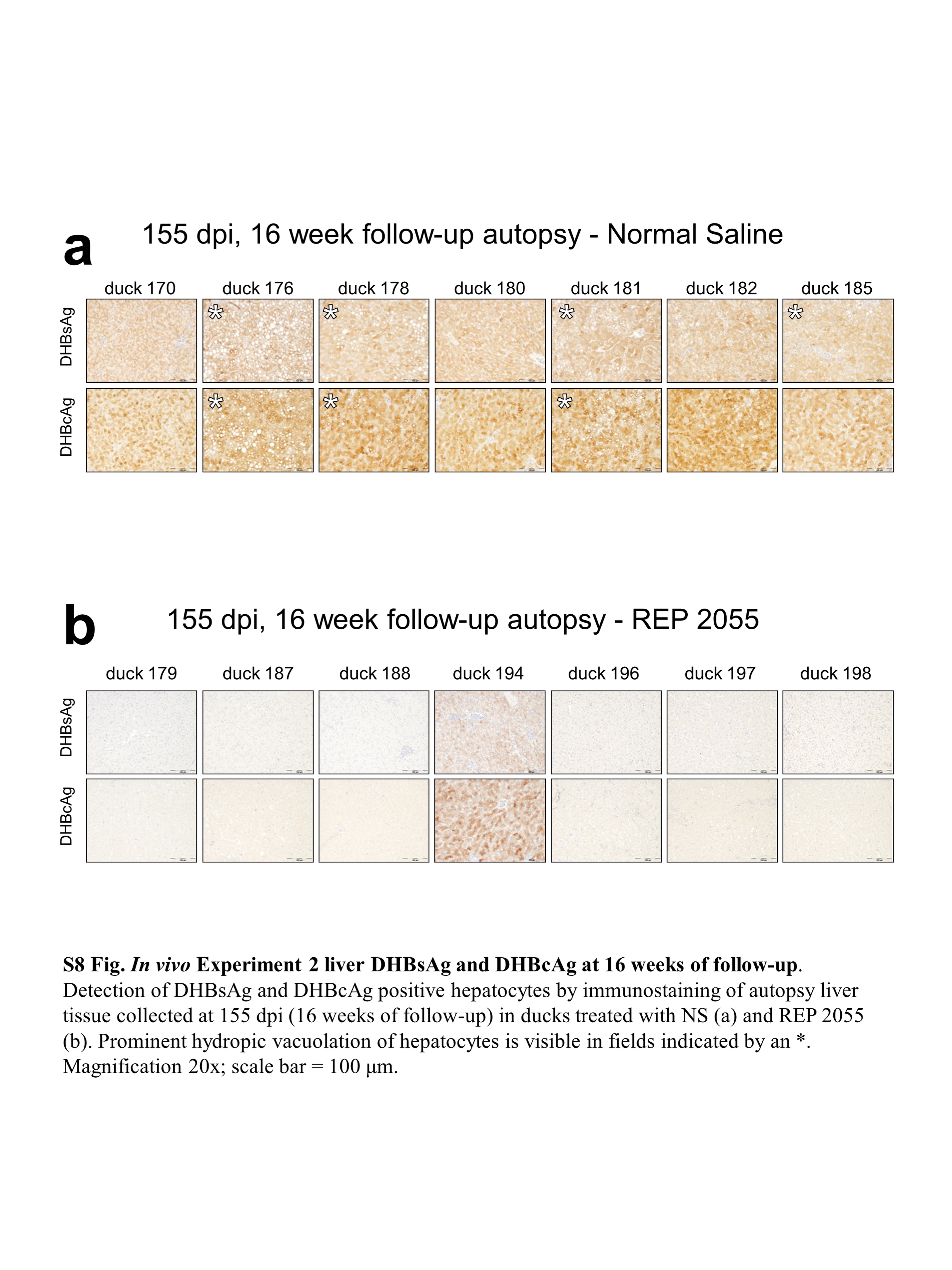

Supplement: S8 Fig — Detection of DHBsAg and DHBcAg positive hepatocytes by immunostaining of autopsy liver tissue collected at 155 dpi (16 weeks of follow-up) in ducks treated with NS (a) and REP 2055 (b). Prominent hydropic vacuolation of hepatocytes is visible in fields indicated by an *. Magnification 20x; scale bar = 100 μm. (TIF) [file pone.0140909.s008.tif]
